# Patient acceptability of targeted risk-based detection of non-communicable diseases in a dental and pharmacy setting

**DOI:** 10.1186/s12889-020-09649-7

**Published:** 2020-10-20

**Authors:** Zehra Yonel, Asma Yahyouche, Zahra Jalal, Alistair James, Thomas Dietrich, Iain L. C. Chapple

**Affiliations:** 1 The Periodontal Research Group, School of Dentistry University of Birmingham, 5 Mill Pool Way, Birmingham, B5 7EG UK; 2 School of Pharmacy, University of Birmingham, Birmingham, UK

**Keywords:** Non-communicable diseases, Screening, Prevention, Dental, Pharmacy

## Abstract

**Background:**

Non-communicable diseases [NCDs] are the major cause of mortality globally and are increasing in prevalence. Different healthcare professionals’ access different population groups; and engaging allied healthcare professionals in risk-driven early case detection of certain NCDs may be beneficial, especially those who have not been tested for NCDs within the previous 12 months.

The objectives of this study were to determine: whether NCD case finding in dental/community pharmacy settings is feasible in terms of patient acceptability, barriers to recruitment, impact on the existing service. Determine time taken to test for: type 2 diabetes risk [T2DM], chronic obstructive pulmonary disease [COPD], hypertension, vitamin D deficiency and chronic kidney disease [CKD]. Determine whether there is added benefit of point of care testing [POCT] to identify diabetes risk compared to a validated screening questionnaire alone.

**Methods:**

An exploratory study was undertaken to explore issues associated with NCD assessment in one dental practice and one community pharmacy within the West-Midlands, UK. Fifty patients > 40 years-of-age were recruited per site. Participants undertook: a questionnaire providing demographic data, any previous NCD diagnosis or positive family history. Validated questionnaires for determining NCD risk [T2DM/COPD]. Chair-side capillary blood [finger-prick] samples for HbA1C, creatinine/eGFR, Vitamin-D.

Prior work had been undertaken to measure the agreement between point of care testing [POCT] devices and a central laboratory method, and to gauge the opinions of participants regarding discomfort experienced using venous (antecubital fossa) and capillary (finger-prick) blood collection, via a 10 cm Visual-Analogue-Scale. The POCT devices demonstrated good concordance with laboratory testing and were acceptable methods of blood collection for participants.

**Results:**

Recruitment rates demonstrated that 8 days were needed to recruit 50 participants and 60% of those approached opted to participate. The principal barrier to participation was time, with average time taken to test being 19mins. Utilising dental and pharmacy settings identified potential cases of previously undiagnosed disease.

**Conclusions:**

Risk-targeted testing for NCDs in high street dental and community pharmacies is both attractive and acceptable to patients.

## Background

The prevalence of chronic non-communicable diseases [NCDs] is increasing and their impact on the global disease burden and healthcare economy is substantial. Evidence in 2015 suggested that 92% of older adults have at least one NCD and 77% have two NCDs [1]. The reason for the increasing prevalence of NCDs is, in part, the result of an ageing population, and also due to an increase in the prevalence of risk-factors common amongst most NCDs such as sedentary lifestyles, refined diets and overweight/obesity. In addition to the substantial health burden, risk-factors for NCDs also contribute a significant economic burden, accounting for over 45% of total NHS costs in the UK in 2006–2007, at approximately £43-billion [2].

Allied healthcare professionals in the UK access large proportions of the population who frequently do not access general medical practice [GP] services [3]. Given the growing NCD burden, this study aimed to determine patient acceptability and potential barriers to utilising allied healthcare professionals such as dentists and pharmacists in order to assist GPs with the NCD epidemic, through targeted risk-based assessment and early detection.

### Rationale for risk directed early NCD detection in dental practice and community pharmacy settings

#### Dental

Members of the public usually only attend their GP when they are unwell, whereas, many people routinely visit their dentists on a regular (6–12 monthly) basis, thus facilitating prevention and lifestyle interventions [4]. Evidence from the USA suggests that, in 2008, 24% of people did not have contact with a general healthcare provider, yet 23% of those accessed a dentist during that time [5]. This was also reported for a UK population, where 12% of patients who reported seeing a dentist biannually reported they had not had contact with a GP in the same 12-month period [3]. Furthermore, 48% of those who reported being regular dental attenders advised having never had a health check at their GP surgery [3]. With approximately 60% of the UK population registered with a dentist [6], this places dental teams, with access to patients who would not necessarily attend their GP regularly, in an ideal position to target patients for risk assessments.

#### Pharmacy

The 2011 Pharmaceutical Group of the European Union survey reported that 98% of European patients can reach their nearest community pharmacy within 30 min, while 58% indicated that their closest community pharmacy was within 5 min of their home. This may render pharmacy settings ideal for early identification of NCDs and provision of preventative advice for large population groups, who may not routinely have access to other healthcare professionals. In addition, over the past four decades there has been a move in pharmacy practice away from the traditional focus on dispensing towards a more patient-centred clinical role [7]. United Kingdom [UK] policy and pharmacists’ professional organisations have stressed the potential of community pharmacists to extend their roles in patient care services to include screening for NCDs. This has been emphasised in policy papers calling for a wider use of community pharmacists in primary patient care [8–10].

#### Inter-professional collaboration

The development of government policies and guidelines advocating the role of allied healthcare professionals in risk-assessment, prevention programs and risk identification for NCDs, suggests that a collaborative approach to tackle the growing NCD burden is required [11]. It is currently common for dentists to liaise with GPs in relation to medications a patient may be taking, especially where these may have an impact on oral health, such as calcium channel blockers which may result in gingival overgrowth. Dentists also work closely with a patient’s medical team when the dentist suspects underlying conditions based on the oral manifestations of systemic diseases. One such example is poorly controlled type 2 diabetes [T2DM]. T2DM may present with oral signs and symptoms including multiple lateral periodontal abscesses. Recently the International Diabetes Federation and European Federation of Periodontology produced joint guidelines for medical and dental professionals for the effective management of patients with periodontitis and, or T2DM in recognition of the strong associations between oral and systemic health [12].

Community pharmacists play an important role in delivering public health services for example vaccinations, health checks, smoking cessation and weight management to complement GP roles. In addition to pharmacist role in optimising the use of medicines in liaison with GPs, providing advice about safe and effective use of medicines when dispensing to patients with prescriptions for the treatment of diabetes, heart disease and hypertension and thus relieving the pressure on the GP practices and A&E. Furthermore, pharmacists work directly in general practice as part of the multi-disciplinary team, in patient facing roles when managing conditions such as diabetes and hypertension [13]. A recent systematic review and meta-analysis which included 21 RCTs (8933 patients) showed that pharmacists-led interventions, as part of a team in general practice, can significantly reduce medical risk factors of CVD events when managing patients with hypertension, diabetes and dyslipidaemia [14].

#### Risk-assessments

Risk-assessment strategies need to ideally provide high sensitivity and specificity so we can discriminate between those who truly do and do not have the condition, be acceptable to patients undergoing assessment, acceptable to the professional delivering the assessment and also demonstrate cost-effectiveness. Venous blood samples are often considered the “gold-standard” testing method for diagnosing many NCDs. The feasibility of primary care dental teams and community pharmacies undertaking venous blood sampling to assess for NCDs is low – as this is not within their routine scope of practice, in addition to the time and resources required to test in this way. Alternative methods for undertaking risk-assessments were considered in this study including the use of validated risk-assessment questionnaires and point-of-care testing [POCT] devices.

Validated questionnaires may be effective ways of stratifying the population into risk groups to allow more invasive and costly tests to be targeted to those in the population most in need. Though, the identification of “at risk” individuals with risk-assessment questionnaires are often satisfactory they often have lower sensitivity and specificity than conventional testing methods. But this has to be weighed up against the advantages of ease of testing, patient acceptability and relatively low associated costs. Given that the aims of risk-assessment in primary care dental and pharmacy settings are not to formally diagnose but to indicate those who may be at elevated risk, the reduction in accuracy may be acceptable given the aforementioned advantages.

POCT remains controversial due to the historical challenges associated with a wide range of devices available, each with their own advantages, disadvantages and varying levels of accuracy [15, 16]. However, the improved quality of POCT devices for capillary blood sampling has resulted more recently in NICE and other national bodies recommending their use for diagnosis of certain NCDs [15, 17–20]. Given that we are not proposing primary care dental teams and community pharmacists formally diagnose, but instead identify those who may be at risk and require further management, they may be ideal for the purpose of risk-assessment in primary care and community settings. The relative ease of use, the near immediate results and the reported patient satisfaction related to POCT are also advantageous. However, it is important that practitioners are aware of the limitations associated with their specific device and the cost associated with these devices may be higher than conventional testing methods.

Patient acceptability of undertaking targeted risk-based detection for NCDs in UK dental and pharmacy settings is currently unknown and requires further investigation. Therefore, an exploratory study was undertaken within one dental practice and one community pharmacy within the West-Midlands, UK, to determine patient acceptability of risk-assessment for NCDs in these settings.

### Aims and objectives

The overarching aim of this study was to assess patient acceptability of screening for NCDs in a primary dental care and a community pharmacy setting. Further objectives of the study included:

1. To identify whether testing for NCDs in a high street dental practice and a community pharmacy setting was feasible in terms of logistics, environment and process. Including feasibility of participant recruitment and barriers to recruitment.
2. To determine whether there is benefit to the finger prick HbA1C test to identify diabetes risk compared to a validated screening questionnaire alone.
3. To ascertain changes needed in the study protocol and barriers to a larger scale study.
4. To determine whether any patients potentially at high-risk of NCDs could be identified where disease status was previously unknown.

## Methods

One dental practice serving both National Health Service (NHS) patients and private patients was selected for participation in the study. Only those patients attending the practice for provision of NHS dental services were approached for participation in the study. The dental practice was situated in the West Midlands, as was the Community Pharmacy. Screening was undertaken for 50 consecutive patients recruited at each site.

Patients over 40 years of age were given a patient information leaflet (PIL) and consented to participate in the study, and a member of the research team conducted the screening as per the standard operating protocol (SOP) (Additional file 1: Appendix 1). A recruitment log was completed, as was any reason cited for non-participation. Time taken to complete the process from consent to completion was also recorded. Participants completed a questionnaire outlining demographic data and previous diagnosis and family history of NCDs. Upon completion of the risk-assessment process participants were also asked to provide feedback or additional comments related to the risk-assessment process (Additional file 1: Appendix 2).

Validated risk-assessment questionnaires were undertaken for determining participants’ risk of T2DM [21] and chronic obstructive pulmonary disease [COPD] [22]. The “Diabetes Risk Score” developed by Leicester University and Diabetes UK is a validated tool recommended by NICE. The risk-assessment consists of seven questions giving a score between 0 and 47. Depending on the patients total score they are categorised into one of four groups: low risk, increased risk, moderate risk or high risk. The risk assessment gives both the current risk of having undiagnosed T2DM, but also a 10 year risk of developing the condition [21]. The COPD risk score “Drive4COPD” is also a validated tool. This risk-assessment consists of five questions resulting in a score from 0 to 10. Depending on the total score awarded patients are then categorised into one of two groups those with a total score that is greater than or equal to 5 or those with a score less than 5 [22].

POCTs to ascertain the presence or absence of risk-factors for the following NCDs: T2DM (HbA1c capillary blood sample), hypertension, atrial fibrillation [AF], height and weight (BMI calculation) - as surrogate markers for cardiovascular disease [CVD], chronic kidney disease [CKD] (creatinine and eGFR capillary blood sample) and Vitamin-D deficiency (capillary blood sample).

### Inclusion and exclusion criteria

#### Inclusion criteria

1. Be able to provide informed consent to participate in the trial
2. Patients aged ≥40 years
3. treatment via NHS services

### Exclusion criteria

1. Not meeting the inclusion criteria or
2. Not amenable to proposed testing method i.e. finger-prick testing.
3. Solely private patients

As part of screening the participant undertook:

1. A questionnaire to provide basic demographic data and to ascertain any previous diagnosis of any of the NCDs, or a positive family history of any of the NCDs. (Additional file 1: Appendix 2)
2. A validated questionnaire to determine risk of COPD [Drive4COPD] [22].
3. A validated questionnaire for risk of diabetes [Leicester Risk Assessment Tool, Diabetes Know your risk] [21].
4. Blood pressure, pulse and AF monitoring using the National Institute for health and Care Excellence [NICE] approved WatchBP device.
5. A chair-side finger-prick sample to assess HbA1C concentration/levels [Siemens/Bayer DCA Vantage]
6. A chair-side finger-prick sample to assess eGFR [Nova StatSensor]
7. A chair-side finger-prick sample to assess vitamin-D levels [Cityassays.org.uk]

Two methods were utilised to risk assess for non-diabetic hyperglycaemia (NDH) and T2DM. The “Leicester Risk-Assessment” questionnaire [LRA] tool, which is validated and recommended by NICE and Diabetes UK; and a point-of care HbA1c test (DCA-Vantage, Siemens).

All analyses were performed by trained members of the research team and the logistics and time involved recorded alongside patient feedback.

Initial data was analysed to establish answers to the research questions. Accepted reference values were used based on current UK guidelines for each of the specific conditions assessed.

### Recruitment process

In each setting a consecutive sampling approach was adopted with potential participants identified by a member of the study team who applied the inclusion/ exclusion criteria and if eligible, written informed consent for their participation in the trial was obtained. Recruitment continued until the recruitment target of 50 participants was met, refusal rate was recorded and if participant was willing to disclose reason for refusal this too was documented.

Those participants for whom an abnormal finding or presumptive diagnosis was identified were advised to visit their GP and a follow-up letter was forwarded to their GP, with participant consent. Only if the participant did not provide consent for their GP to be contacted was a general letter with their results of interest provided to the participant such that they could present it to their GP at a later date should they so desire.

### Statistical analysis plan

Descriptive statistics were used to analyse the study findings. Data on the recruitment to the study was also analysed descriptively, including the number of patients approached, the number that agreed to participate and number eligible to participate. Reasons for non-entry into the study were assessed. Reasons for non-completion were also analysed descriptively.

No formal sample size calculation was undertaken for this study. The sample size (*n* = 50, in each site) was deemed sufficient to enable identification of practical challenges involved with running such a study in a dental and pharmacy setting and allowed identification of areas where change is required prior to implementing such a model on a larger scale. The intention of this phase of the study was to identify barriers to conducting a similar style study using a larger sample within multiple primary care dental practices and pharmacies, across a broader geographical area. This study was not powered to detect new cases of disease.

## Results

Table 1 demonstrates that the study was balanced for males and females in both settings. There was a spread across each of the age categories with the average age of participants in dental settings being younger than those recruited in pharmacy settings, with a mean age of 58 years and 65 years respectively. In the dental setting all participants identified themselves as white/Caucasian and in the pharmacy setting all but 1 participant identified themselves as white/Caucasian. Most participants reported themselves to be retired with the second highest category being professionals in both settings. There were approximately three times as many professionals in the dental setting compared to the pharmacy setting (32:11) and more participants in the pharmacy setting considered themselves to be in manual or non-manual work. In both dental and pharmacy settings about half of participants considered themselves non-smokers who had never smoked (52% & 51% respectively) (Additional file 1: Appendix 3), with approximately a third of participants reporting being previous smokers (38% & 32% respectively) in both settings (Table 1).

**Table 1:** Summarising demographic data of participants recruited from dental and pharmacy settings

	Dental	Pharmacy
Number recruited (N)	50	51
Time taken to recruit (days)	8	14
% Female	47	53
% Age category:		
40–49	18	10
50–59	37	20
60–69	29	30
70+	16	40
% Occupation:		
Unemployed	0	4
Manual	14	10
Non-Manual	2	10
Executive/Managerial	8	4
Professional	32	11
Retired	44	61

### Recruitment and impact on existing service

There was a 60% conversion rate in the dental setting and the recruitment target of 50 participants achieved in 8 days. Recruitment in the pharmacy setting showed a 59% conversion rate and the recruitment target of 50 participants achieved in 14 days. The main reason cited for declined participation in both settings was time. In addition to being the major barrier to recruitment, time was also the major consideration when determining impact on existing services. The average time taken for case-detection in both settings was 19mins.

### Demographic data

The most common age category sampled within the dental setting were participants between the ages of 50–59 years followed by the 60–69 years category. The mean age was 58 years with the oldest participant being aged 89 years and the youngest aged 41 years. The most common age category sampled within the pharmacy setting were participants aged 70+ years followed by the 60–69 years category. The mean age was 65 years with the oldest participant being aged 83 years and the youngest participant aged 40 years.

Female participants made up 47% of the dental sample and all participants in the dental setting identified their ethnicity as white/Caucasian. 44% of volunteers were retired, 32% considered themselves to be a professional, 14% were manual workers, 2% were non-manual workers and 8% considered themselves to be executive/managerial workers (Table 1). Female participants made up 53% of the pharmacy sample and all participants except 1 in the pharmacy setting identified themselves to be White/Caucasian ethnicity. 61% of patients were retired, 11% considered themselves to be a professional, 10% were manual workers, 10% were non-manual workers and 4% considered themselves to be executive/managerial workers (Table 1).

### Diabetes

**Fig. 1:**
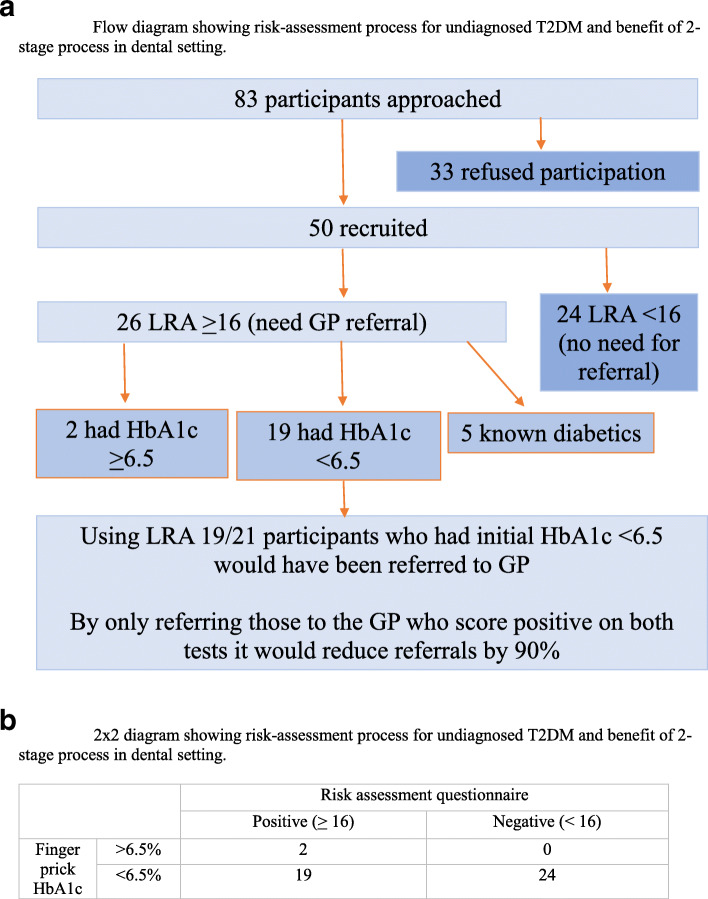
**a** Flow diagram showing risk-assessment process for undiagnosed T2DM and benefit of 2-stage process in dental setting. **b** 2 × 2 diagram showing risk-assessment process for undiagnosed T2DM and benefit of 2-stage process in dental setting

In the dental setting of 45 patients without an existing diagnosis of diabetes, 21 (47%) rated high-risk on the LRA, the recommendation for which is GP referral. Of these 2 (4.4%) had an HbA1c in the diabetes range (> 48 mmol/mol). A further 7 (16%) had scores 42-48 mmol/mol (NDH). However, 12/21 who were highlighted as in need of referral to a GP according to the LRA, actually had a HbA1C within the healthy reference range (< 42 mmol/mol) (Fig. 1a and b).

In the pharmacy setting of 44 patients without an existing diagnosis of diabetes, 13 (30%) rated high-risk on the LRA, with a further 13 (30%) rated moderate risk, the recommendation for which is GP referral. Of these, 4 had an HbA1c in the diabetes range (> 48 mmol/mol) A further 7 had scores between 42 and 48 mmol/mol (NDH). In the pharmacy setting a total of 26 participants were highlighted as needing referral to GP according to the LRA, with only 11 having a HbA1C greater than 42 mmol/mol according to the POCT. One participant who had a finger prick HbA1c in the NDH range was flagged in the increased risk category (which according to the LRA does not require referral to GP but advises lifestyle changes to be made).

### CVD

In the dental setting 34% of participants were deemed to be overweight based on their BMI with a further 28% having a BMI greater than 30 classifying them as obese. Of those who stated they did not believe themselves to have CVD or hypertension, 17 (44%) had an elevated systolic reading (> 140 mmHg) and 13% had a diastolic reading > 100 mmHg.

In the pharmacy setting 47% of participants were deemed to be overweight based on their BMI with a further 25% having a BMI greater than 30 classifying them as obese. Of the 26 participants who stated they did not believe themselves to have CVD or hypertension, 9 (41%) had an elevated systolic reading (> 140 mmHg) and 35% had a diastolic reading > 100 mmHg.

### CKD

Only one participant in the dental setting stated they had known chronic kidney disease when asked. Although most participants had an estimated glomerular filtration rate (eGFR) > 90, 11 participants had an eGFR of 89–60 (stage 2 kidney disease) and a further 4 had an eGFR of between 55 and 49 (stage 3a kidney disease).

Only one participant in the pharmacy setting stated they had known chronic kidney disease when asked. Yet, although 16 participants had an eGFR > 90, 19 participants had an eGFR of 89–60 and a further 6 had an eGFR of between 55 and 49.

### COPD

Two participants in the dental setting reported knowing they had COPD. In addition to correctly identifying those 2 participants the COPD risk assessment tool also highlighted a further 2 participants in the dental setting who may be at increased risk of COPD.

The COPD risk assessment tool identified 7 people who may be at increased risk of COPD in the pharmacy setting. Three participants in the pharmacy setting reported knowing they had COPD, of which 1 participant was picked up by the risk assessment tool as being high risk while the other 2 were missed. A further 6 participants who thought themselves not to have COPD were identified by the risk assessment tool as being high risk and in need of referral to a GP.

### Vitamin D

In the dental setting 8 participants were highlighted as having insufficient vitamin D levels, none of whom were aware of having vitamin D insufficiency. Of the three participants who reported thinking they were deficient in vitamin D, all had results within the healthy reference value.

In the pharmacy setting 7 participants were highlighted as having insufficient vitamin D (30.1-50 nmol/L) and a further 2 were deficient (15-30 nmol/L), none of whom were aware of having vitamin D insufficiency/deficiency. Of the 2 participants who reported thinking they were deficient in vitamin D, all had results within the healthy reference range.

### Patient acceptability

Of those subjects who participated acceptability and satisfaction was very positive with only 3 participants providing neutral or negative feedback (Additional file 1: Appendix 4). Of those patients who declined participation no additional feedback was received except for reason for refusal, the most common being a lack of time.

## Discussion

This overarching aim of this study was to assess patient acceptability of screening for NCDs in primary care dental practices and community pharmacy settings, with a view to determine practical challenges and barriers relating to logistics, environment and process, whether there was benefit to POCT testing HbA1c in addition to risk-assessment tool alone and to ascertain barriers to a larger scale study. A further objective was to determine whether potentially high-risk of NCDs could be identified within these settings where individual risk or disease status was previously unknown.

Recruitment rates were better in a dental setting with half the amount of time required to reach the recruitment target of 50 participants. However, the time take to recruit participants in both settings was satisfactory with no obvious recruitment challenges experienced by the study team. However, it must be noted that although the participants enrolled in the study were of a range of ages and a satisfactory gender balance. The study participants were not representative of the general population of the West Midlands in terms of ethnic identity. Thus it remains to be determined whether recruitment of individual’s from ethnic backgrounds known to be associated with increased risk of these specific NCDs is achievable based on the results of this study.

The demographic data for patients in both the dental and pharmacy settings were comparable although more people identified themselves as professionals within the dental than the pharmacy setting. In both the dental and pharmacy setting the patient satisfaction and acceptability was high. Participants found the method of testing acceptable and participant feedback relating to testing for NCDs in both dental and pharmacy settings was positive (Table 2).

The main reason cited for non-participation in both settings was lack of time. The average time taken to test in both settings was 19mins. Where an additional member of staff was not available to undertake risk-assessment the potential impact on routine activities in both settings would be significant with increased delays. In the dental setting patients arriving for appointments often attended the practice in advance of the scheduled appointment time, thus could be offered a risk assessment prior to seeing the dental team or could be offered a risk-assessment immediately upon completion of their dental appointment. In this particular practice there was a spare surgery available for the risk-assessment to take place. However, where an additional room was not available increased waiting time and potential delays to risk-assessments or scheduled dental activity may pose an additional barrier. The key finding relating to impact on current service was therefore for risk-assessment to be undertaken effectively an additional member of dedicated staff would be required to undertake testing and an additional room dedicated to the risk-assessment process.

The benefit of undertaking a two-step risk-assessment process for identifying potential T2DM was shown to be beneficial in improving the specificity of the T2DM risk-assessment (Fig. 1a). When questionnaire based risk-assessment alone was used it resulted in potentially 90% more referrals to GP than when a two-step risk assessment process was utilised. Clearly given how busy GP colleagues are and the time burden they are already under caring for patients, it is important that their time is protected and not taken up by inappropriate, unnecessary referrals. Thus a two-stage risk-assessment would appear preferable, however a full economic evaluation comparing these methods has not been undertaken.

The risk-assessment methods used appeared to identify people at high risk of NCDs who were previously undiagnosed and unaware of their risk status. Potential cases of previously undiagnosed disease were identified in both dental and pharmacy settings. This is despite the fact that the demographic of the study population predominantly identified as “White/Caucasian” and of higher socio-economic status; not being the groups conventionally considered as being highest risk for developing NCDs. Further research to determine whether the findings are also applicable in groups commonly considered of higher-risk and also research to follow-up patients to determine how many go on to receive formal diagnosis and onward management is needed to understand the true potential impact of risk-assessment for NCDs in these settings.

The main challenges associated with the study include the sample size employed, this was small as the purpose of the study was to demonstrate patient acceptability and potential barriers prior to undertaking a formal feasibility study for a definitive trial. The study was not-representative of the population with almost 100% of participants identifying themselves as white/Caucasian. Whilst we demonstrated that testing in these locations can be undertaken to good effect when a dedicated member of staff is undertaking the risk-assessment process, this may not be possible in everyday practice where an additional staff member may not always be available. In the pharmacy and dental settings, the additional service was logistically challenging alongside traditional duties when no additional staff were available to undertake the risk-assessments associated with the study. Additionally, securing funding at the individual pharmacy and dental practice to provide such services could act as a barrier. Further work is needed to demonstrate that this can be done by the existing team within each setting and to demonstrate the cost-effectiveness of the risk-assessment process should an additional dedicated member of staff be required.

To our knowledge this method of risk-assessing for multiple NCDs in a dental setting has not previously been undertaken. Utilising dental settings to test for T2DM has been demonstrated to good effect outside of the UK and this study further supports those findings [23–27]. We also demonstrated the advantage of a 2-step risk-assessment process for T2DM which is supported by the study of Bould et al. [28]. Similarly, in a pharmacy setting isolated small-scale pilot initiatives have shown promising results, but nationally POCT and risk assessment for multiple NCDs is not standard practice. Although small initiatives for screening for NCDs have been undertaken in UK pharmacies, besides the NHS Health Check (which is a health check-up designed to spot early signs of kidney disease, heart disease and type 2 diabetes) and The Healthy Living Pharmacies (HLPs) initiative very few services in UK pharmacies have been consistent. This is despite these being part of the NHS Long Term Plan, therefore, this study could add to the existing evidence and support prevention roles for pharmacists [29, 30].

NICE currently recommends that allied healthcare professionals, including community pharmacists and general dental practitioners [GDPs], should risk-assess for T2DM [31]. To the authors knowledge this is not currently undertaken in general dental practice nor is it routine practice in community pharmacies at a national level. Furthermore, the feasibility of such risk-assessments has yet to been determined. Our study provides the groundwork for investigating this further, having determined a positive response from patients accessing these services and that the potential devices required to undertake the risk-assessments perform well. This study demonstrated strong support from participants for the use of allied healthcare professionals to provide targeted risk-assessments for NCDs. It also demonstrated that the methods required to undertake such assessments were acceptable to participants.

However, the concept of dentists and pharmacists testing for NCDs is not without controversy. Firstly, the UK National Screening Committee clearly states that it does not support population-based screening for NCDs [32]. Though evidence suggests a population-based screening programme lacks benefit, the potential benefits of an opportunistic risk-directed assessment of patients who have risk-factors for NCDs, and who may not have had contact with another healthcare professional in the proceeding 12 months is yet to be determined. Opticians currently identify potential signs of CVD and T2DM and advise patients to seek GP follow-up and refer patients to their GP for formal assessment. The present study provides insights into the potential for a similar approach in high street dental surgeries and community pharmacies. Further work is needed to determine feasibility of such a model within the UK healthcare system to assess both the effectiveness and cost-effectiveness of such a strategy and to ensure suitable care pathways for those patients identified with new cases of disease are accessible.

Before further larger scale studies can be undertaken to determine cost-effectiveness and clinical effectiveness of undertaking such targeted risk assessment’s in dental and pharmacy settings, careful consideration must be given to the patient’s care pathway following identification of a previously unknown elevated risk status. Moreover, care must be taken to avoid duplicated testing as many patients may have already undergone a NHS Health-check with their GP in the previous 12 months. In addition, care and consideration is required to prevent adding to the ever-growing burden on GPs by increased referral loads without consideration of how these patients should be managed and how the additional referrals will be funded. Further work is needed to determine what the additional burden to Primary Care services could be and to mitigate for this, whilst also assessing the health economic impact of such an approach.

## Conclusion

Although there is controversy surrounding the precision and accuracy of POCT, the devices tested in this study demonstrated good levels of concordance with standard laboratory methods and may present a viable alternative to laboratory-based methods when risk-assessing patients for NCDs in community settings. Participant acceptability to finger-prick testing was positive. Further work is required to determine whether testing for NCDs in a dental practice and pharmacy setting is feasible in terms of logistics, environment and process. Based on this work it appears that to minimise the negative impact on day-to-day running of current services additional dedicated staff may be required to undertake the risk-assessment in dental and pharmacy settings. Further work also needs to be undertaken with suitable follow up to determine whether there are health and economic benefits to such a model.

## Supplementary information

**Additional file 1: Table** **1****.** Sample of positive feedback and all neutral and negative feedback from participants in dental and pharmacy settings. **Table 2.** Summarising demographic data of participants recruited from dental and pharmacy settings.

## Data Availability

The datasets used and/or analysed during the current study are available from the corresponding author on reasonable request.

## Abbreviations

NCDs: Non-communicable disease
POCT: point of care testing
eGFR: estimated Glomerular-Filtration-Rate
CVD: Cardiovascular disease
T2DM: Type-2 diabetes mellitus
CKD: Chronic kidney disease
COPD: Chronic obstructive pulmonary disease
NICE: National Institute for health and Care Excellence
GDPs: General dental practitioners
NDH: Non-diabetic Hyperglycaemia (Prediabetes)
PIL: Patient Information Leaflet
VAS: Visual analogue score
SOP: Standard operating procedure
BDH: Birmingham’s Dental Hospital
AF: Atrial fibrillation
LRA: Leicester Risk Assessment
