# Correction to: Patient acceptability of targeted risk-based detection of non-communicable diseases in a dental and pharmacy setting

**DOI:** 10.1186/s12889-021-10303-z

**Published:** 2021-02-11

**Authors:** Zehra Yonel, Asma Yahyouche, Zahra Jalal, Alistair James, Thomas Dietrich, Iain L. C. Chapple

**Affiliations:** 1 The Periodontal Research Group, School of Dentistry University of Birmingham, 5 Mill Pool Way, Birmingham, B5 7EG UK; 2 School of Pharmacy, University of Birmingham, Birmingham, UK

**Correction to: BMC Public Health 20, 1576 (2020)**

**https://doi.org/10.1186/s12889-020-09649-7**

It was highlighted that in the original article [1] the Acknowledgments section was incomplete. This Correction article shows the incorrect and correct Acknowledgments section. The original article has been updated.

**Incorrect**

- Stapenhill Dental Practice and the dental team who facilitated completion of the study – Burton on Trent.
- Knights Pharmacy and the pharmacy team who facilitated completion of the study - Bromsgrove.

**Correct**

- Nova Biomedical - The team at Nova Biomedical generously provided testing equipment and consumables for completion of the project in addition to providing staff training in the appropriate use of all testing equipment.
- Siemens Healthineers – Mr. S Carey, Mr. A Sheppard and the team at Siemens Healthineers who generously provided testing equipment and consumables for completion of the project in addition to providing staff training for the appropriate use of all testing equipment.
- Stapenhill Dental Practice and the dental team who facilitated completion of the study – Burton on Trent.
- Knights Pharmacy and the pharmacy team who facilitated completion of the study - Bromsgrove.

## References

[1] Yonel Z (2020). Patient acceptability of targeted risk-based detection of non-communicable diseases in a dental and pharmacy setting. BMC Public Health.

