# Supplementary material for: Patient acceptability of targeted risk-based detection of non-communicable diseases in a dental and pharmacy setting
Source: BMC Public Health. 2020 Oct 20;20:1576. doi: 10.1186/s12889-020-09649-7 (PMC7576866; doi:10.1186/s12889-020-09649-7)
Supplement: Supplementary file 1 — Additional file 1: Table 1. Sample of positive feedback and all neutral and negative feedback from participants in dental and pharmacy settings. Table 2. Summarising demographic data of participants recruited from dental and pharmacy settings. [file 12889_2020_9649_MOESM1_ESM.zip › 05.10.2020 AppendicesR4.docx]

# Appendix 1

Standard operating protocol: REC reference:17/WM//0022

Protocol number: RG_16-102, IRAS project ID:200232

- Approach potential participant ask if they are willing to hear about a study being undertaken on site.
  - If yes, provide information and provide PIS
  - If no, ask if they would be willing to explain why and record on recruitment log reason for refusal.
- Following information provision, ask potential participant if they require further information, have any questions and determine whether they wish to participate.
  - If yes, proceed to complete written informed consent
  - If no, ask if they would be willing to explain why and record on recruitment log reason for refusal
- Complete written consent process in line with GCP / ethical requirements and study protocol.
- Retain 1 consent for site file and provide an additional consent form for participant to keep.
- Upon obtaining consent, proceed with study, in order outlined on RedCap electronic survey.
  - Study ID, Time and location of screening
  - Demographic data
  - Questions relating to participant history of diagnosed of Non-communicable disease (NCD)
  - Questions relating to participant family history of diagnosed of Non-communicable disease (NCD)
  - Study data collection: - all equipment must only be used by a member of the research team trained in the correct use in accordance with manufactures guidance.
    - Height, weight, Hip, Waist measurements
    - Calculation of BMI
    - Validated Diabetes risk assessment questionnaire
    - Validated COPD risk assessment Questionnaire
    - Blood pressure recording using NICE recommended device
  - Point of care devices – at this stage the participant will be informed that a finger-spot blood sample will be required. They will be asked if they are still happy to proceed with this element.
  - Upon receiving verbal confirmation of consent the standardised lancets are used to produce a blood spot as per manufactures instructions
  - The blood spot is used to provide a sample for the following test
    - DCA Vantage – HbA1c – test time 6mins
    - Nova StatSensor – Creatinine /eGFR – test time 30 seconds
    - CityAssays – Vitamin D – Requires posting to offsite laboratory
  - Where blood sample is insufficient – participant may be requested to provide an additional finger spot sample
- Participant will then answer remaining questions relating to patient acceptability
- Time of assessment completion and whether referral to GP is required will then be recorded.
- On completion of the risk assessment participants will be informed if any readings are outside of expected range and will also be informed that these “tests” are used solely for the purpose of research and need to be interpreted with caution as they are not diagnostic.
- Where results fall outside of expected range consent will be obtained from participant to write to their GP providing information relating to the study and the results obtained.
  - Participant should be informed this may result in the GP making contact to request an appointment.
  - Where participant withholds consent for their GP to be contacted an additional copy of the results with a GP cover letter must be provided to the participant such that they can submit it to their GP should they wish to at a future date.
- Participant will be asked to consent to the provision of their contact details, these will be used in line with data protection policies and the sole purpose of provision of the Vitamin D result which will not be available at time of testing.
- Where appropriate participants will be provided with leaflet on healthy lifestyle choices and signposted to relevant NHS accredited literature.

Appendix 2

Research Data Collection Survey

(see PDF uploaded separately)

Appendix 3

Table 1a: summarising demographic data of participants recruited from dental and pharmacy settings.

|  | **Dental** | **Pharmacy** |
| --- | --- | --- |
| Number recruited (N) | 50 | 51 |
| Time taken to recruit (days) | 8 | 14 |
| % Female | 47 | 53 |
| % Age category: |  |  |
| 40-49 | 18 | 10 |
| 50-59 | 37 | 20 |
| 60-69 | 29 | 30 |
| 70+ | 16 | 40 |
| % Ethnicity: |  |  |
| African | 0 | 0 |
| Bangladeshi | 0 | 0 |
| Caribbean | 0 | 0 |
| Indian | 0 | 2 |
| Pakistani | 0 | 0 |
| Asian Other | 0 | 0 |
| Mixed-race | 0 | 0 |
| White/Caucasian | 100 | 98 |
| Other | 0 | 0 |
| % Occupation: |  |  |
| Unemployed | 0 | 4 |
| Manual | 14 | 10 |
| Non-Manual | 2 | 10 |
| Executive/Managerial | 8 | 4 |
| Professional | 32 | 11 |
| Retired | 44 | 61 |
| % Smoking: |  |  |
| Current | 10 | 16 |
| Previous | 38 | 33 |
| Never | 52 | 51 |
| % BMI: |  |  |
| Overweight | 34 | 47 |
| Obese | 28 | 25 |
| % Previous Diagnosis of: |  |  |
| Periodontitis | 18 | 22 |
| T2DM | 10 | 12 |
| COPD | 4 | 6 |
| CVD | 22 | 45 |
| Vitamin D deficiency | 6 | 4 |
| CKD | 2 | 2 |
| % Known Family History of: |  |  |
| Periodontitis | 20 | 22 |
| T2DM | 34 | 43 |
| COPD | 22 | 18 |
| CVD | 60 | 63 |
| Vitamin D deficiency | 4 | 2 |
| CKD | 4 | 8 |

Appendix 4

Table 1: Sample of positive feedback and all neutral and negative feedback from participants in dental and pharmacy settings.

| **Positive participant feedback from those attending Dental Setting** | **Positive participant feedback from those attending Pharmacy Setting** | **Negative participant feedback from Dental Setting** | **Negative participant feedback from Pharmacy Setting** |
| --- | --- | --- | --- |
| “It’s a good idea to know what risks are coming your way”  Female, aged 44 | “Convenient, opportunistic you have to make time to go to doctor”  Female, aged 62 | “I go to my GP regularly but may be useful for others”  Male, aged 53 | “Can be disruptive if you have to share rooms. But good to pick things up.”  Female, aged 74 |
| “Because you see your doctor less and you might not have symptoms ’til it’s too late”  Female, aged 50 | “Good, brilliant. Why bother the doctor. If others can do this let the doctors deal with treating people who really need a GP appointment, you know who are actually sick”  Male, aged 62 |  | “Pharmacy is ok not sure dentists can cope with testing, although any checks will be good”  Male, aged 70 |
| “Two birds, one stone”  Male, aged 62 | “Free up time in doctors’ surgeries”  Male, aged 66 |  |  |
| “Quite nice that it’s more of a full risk assessment, rather than checking on single things. More holistic rather than just a single test. I've just had a cholesterol reading on its own which isn't too meaningful. We have got ourselves into a society that seems to have given responsibility away. Getting people thinking more about their own health is always a benefit.”  Female, aged 57 | “Very relaxed. These days if I’m worried and go to the doctors it seems rushed. Or a phone appointment which I don't like. This is more personalised than a phone call.”  Female, aged 61 |  |  |
| “I think it's a great idea particularly for men. As women are better at going to the doctors.”  Male, aged 52 | “Cuts out the frustration of trying to get a doctor’s appointment.”  Male, aged 73 |  |  |
| “Very good idea. It’s a right rigmarole trying to book appointments and seeing a nurse, when I could just get everything done in one go at the dentist.”  Female, aged 50 | “Brilliant idea it's nice to know you are getting checked and if there is a problem it gets picked up. Lots of people struggle to get into the doctor.”  Female, aged 63 |  |  |
| “I think it’s brilliant - I come to the dentist more than the doctor - especially when things like diabetes you can walk around and not even know you have it”  Female, aged 51 | “If someone doesn't go to the doctor often like me this is good.”  Female, aged 81 |  |  |
| “Saves another appointment with the doctor it's also very hard to get appointments at the doctors surgery”  Female, aged 67 | “Excellent idea to do this. So many people have diabetes that don't know about it. This could pick it up so they can change their lifestyle.”  Male, aged 80 |  |  |
| “You are here every 6 months anyway. It's not like it's another appointment you have to go to.”  Male, aged 46 | “Information is key once you know you can make the decision about it and seek the necessary lifestyles change or professional”  Female, aged 50 |  |  |
| “It gives you another option as I always put off going to the doctor”  Female, aged 75 | “Hard getting into the doctors”  Female, aged 64 |  |  |
| “If you can have it at the same time more convenient no need for separate appointments”  Female, aged 65 | “Can fit it in at a convenient time. So hard to get an appointment at the GP”  Male, aged 54 |  |  |
| “People don't go to the doctors. If things can be prevented or found out sooner strike while the irons hot”  Male, aged 62 | “Handy for the patients as can pop in when collecting my medications”  Male, aged 47 |  |  |
| “Another opportunity for health promotion”  Female, aged 53 | “Any testing is a good thing, setting does not matter”  Female, aged 63 |  |  |
| “People who may benefit from this may not go to the doctors, but anyone can suffer toothache”  Male, aged 50 |  |  |  |
