# Supplementary material for: Patient acceptability of targeted risk-based detection of non-communicable diseases in a dental and pharmacy setting
Source: BMC Public Health. 2020 Oct 20;20:1576. doi: 10.1186/s12889-020-09649-7 (PMC7576866; doi:10.1186/s12889-020-09649-7)
Supplement: Supplementary file 1 — Additional file 1: Table 1. Sample of positive feedback and all neutral and negative feedback from participants in dental and pharmacy settings. Table 2. Summarising demographic data of participants recruited from dental and pharmacy settings. [file 12889_2020_9649_MOESM1_ESM.zip › Alphabet studyeCRFR4.pdf]

Resize font:

UNIVERSITY OF BIRMINGHAM

Birmingham Community Healthcare NHS
NHS Foundation Trust

Alphabet study

Dear Sir/Madam,

Thank you for agreeing to participate in our study. I would be grateful if you could answer the following questions regarding screening for medical conditions in dental / pharmacy settings

Sincerely,
Zehra Yonel
Clinical Lecturer in Periodontology
School of Dentistry

Study ID:
* must provide value

Time at start of Screening
* must provide value

Location for Screening

- Dental practice
- Pharmacy

reset

Demographic data

	Female	Male	Non Specified Gender	Other
**How would you define your gender**	○	○	○	○

reset

What is your Date of Birth?
* must provide value

DOB

Which of the following best defines your ethnicity?
* must provide value

- African
- Asian -Bangledeshi
- Asian - Indian
- Asian - Pakistani
- Asian - Other
- Caribbean
- Caucasian (White)
- Mixed race
- Other

ethnicity

Which of the occupation groups applies best to you?

If retired please also select the pre-retirement category that applies.
* must provide value

- Unemployed
- Manual worker
- Non-manual worker
- Executive/Managerial
- Professional
- Retired

Occupational status

Do you smoke tobacco?
* must provide value

- Yes - currently
- No - previous smoker
- No - Never smoked

reset

Smoking

Have you ever been diagnosed with the following?

	Yes	No	Unsure
**Periodontitis (gum disease)** * must provide value	○	○	○

reset

**Diabetes** * must provide value	○	○	○	reset
**Chronic obstructive pulmonary disease (COPD)** * must provide value	○	○	○	reset
**Cardiovascular disease / hypertension (heart disease / high blood pressure)** * must provide value	○	○	○	reset
**Vitamin D deficiency** * must provide value	○	○	○	reset
**Chronic kidney disease** * must provide value	○	○	○	reset

Has a member of your immediate family (parent / sibling) been diagnosed with one of the following conditions?

	Yes	No	Unsure	
**Periodontitis (gum disease)**	○	○	○	reset
**Diabetes**	○	○	○	reset
**Chronic obstructive pulmonary disease (COPD)**	○	○	○	reset
**Cardiovascular disease / hypertension (heart disease / high blood pressure)**	○	○	○	reset
**Vitamin D deficiency**	○	○	○	reset
**Chronic kidney disease**	○	○	○	reset

Point of care results

Height in centimetres
* must provide value

Expand

Weight in kilograms
* must provide value

Expand

Hip
* must provide value

waist
* must provide value

Body Mass Index

Expand

Leicester University Diabetes risk Score Result (number)
* must provide value

COPD Risk Score (number)
* must provide value

Blood pressure reading
* must provide value

Expand

AF
* must provide value

Positive reading Negative reading

reset

DCA Vantage HbA1C Result
* must provide value

Nova StatSensor eGFR reading
* must provide value

Vitamin D Level

Point of care testing

	Yes	No
**Was the method of collecting the blood samples acceptable?**		

reset

Do you feel screening in this setting is beneficial?

Yes No

reset

Do you feel receiving preventative advice for medical conditions while in this setting is beneficial?
* must provide value

Yes No

reset

Optional feedback from participant

Time that screening is completed
* must provide value

referral to GMP required
* must provide value

Yes No

reset

IRAS ID:
Version: 1,0
Date: 18/07/16

Submit

Powered by REDCap
